# Inhibition of PFKFB3 in Macrophages Has a Dual Effect on Tumor-Regulating Lipid Metabolism

**DOI:** 10.3390/ijms27010217

**Published:** 2025-12-24

**Authors:** Elena Shmakova, Tatiana Sudarskikh, Kseniia Shalygina, Vitaliy Chagovets, Natalia Starodubtseva, Alisa Tokareva, Anastasia Novoselova, Vladimir Frankevich, Anna Tarasova, Dmitry Kostromitsky, Alexey Dobrodeev, Sergey Afanas’ev, Irina Larionova, Julia Kzhyshkowska

**Affiliations:** 1 Laboratory of Translational Cellular and Molecular Biomedicine, National Research Tomsk State University, 634050 Tomsk, Russia; kazakova.e.o@mail.ru (E.S.);; 2 Laboratory of Molecular Therapy of Cancer, Cancer Research Institute, Tomsk National Research Medical Center, Russian Academy of Sciences, 634014 Tomsk, Russia; 3 National Medical Research Center for Obstetrics, Gynecology and Perinatology Named after Academician V.I. Kulakov, Ministry of Healthcare of Russian Federation, 117997 Moscow, Russiavfrankevich@gmail.com (V.F.); 4 Moscow Center for Advanced Studies, 123592 Moscow, Russia; 5 Laboratory of Translational Medicine, Siberian State Medical University, 634050 Tomsk, Russia; 6 Abdominal Department, Cancer Research Institute, Tomsk National Research Medical Center, Russian Academy of Sciences, 634014 Tomsk, Russia; 7 Institute of Transfusion Medicine and Immunology, Institute for Innate Immunoscience (MI3), Medical Faculty Mannheim, Heidelberg University, 68167 Mannheim, Germany

**Keywords:** colorectal cancer, tumor-associated macrophages, PFKFB3, lipid metabolism, amino acid metabolism

## Abstract

Colorectal cancer is the third most common cancer worldwide, making lymph node recovery critical for treatment decisions and prognosis. Within the colorectal tumor microenvironment, the metabolic programming of tumor-associated macrophages (TAMs) can drive both pro- and anti-tumor responses, yet the specific glycolytic pathways governing their pro-metastatic conversion present promising therapeutic targets. This study investigated the role of glycolysis activating enzyme 6-phosphofructo-2-kinase/fructose-2,6-biphosphatase 3 (PFKFB3) in mediating TAMs metabolic polarization, and its potential as a therapeutic target. PFKFB3 expression was found to be predominant in TAMs in CRC tumor samples. Lipidomic analysis performed by HPLC-MS/MS revealed that PFKFB3 inhibition altered glycerophospholipid metabolism (*p* = 6.13 × 10^−10^), and shifted TAMs toward sphingolipid-mediated immunosuppressive metabolism. PFKFB3 activity was associated with a specific reduction in asparagine availability, potentially pointing to a targeted reprogramming of amino acid metabolism supporting distinct TAM functions under conditions of intra-tumoral metabolic stress. These findings highlight PFKFB3 as an essential regulator of TAMs pro-tumoral metabolism in CRC, particularly in colon cancer.

## 1. Introduction

Tumor-associated macrophages (TAMs) are potent innate immune cells that can support all stages of solid tumor initiation and progression, including proliferation benefits of cancer cells, cancer cell migration and immune escape, tumor angiogenesis, extravasation of cancer cells into blood and lymphatic circulation, finding favorable accommodation in a metastatic niche in distant organs or in sentinel lymph nodes [1,2]. The effector mediators produced by TAMs include reactive oxide species (ROS), cytokines, growth factors, chitinase-like proteins, enzymes, and components of the extracellular matrix [2,3,4,5]. Resident TAMs have intrinsic ability to fight with transformed cells, but once cancer cells overcome this barrier, they start to program both resident macrophages and incoming monocyte-derived macrophages to develop tumor-associated TAM phenotype. Selective scavenging of these mediators also enables TAMs to modulate tumor growth control [6]. However, if in the majority of solid cancers TAMs have dominating pro-tumoral activity, in colorectal cancer (CRC), high levels of CD68+ cells correlate with good prognosis [7].

Accumulating data indicate that metabolism of TAMs is principal for their pro-tumor activity [2,8]. If in the acute inflammation macrophages utilize glycolysis for the rapid anti-bacterial response, while homeostatic resident macrophages use fatty acid oxidation allowing slow and economical use of their energy resources, TAMs have mixed metabolism [2,9]. Such mixed metabolism allows TAMs to have sufficient energy for the low-grade production of pro-tumoral mediators for a long time period. There is an urgent need to understand which factors allow TAMs to combine glycolysis with fatty acid oxidation, and how cancer cells can promote low-grade glycolysis in tumor-supporting tolerogenic TAMs.

We have recently identified that expression of the stimulator of glycolysis PFKFB3 is elevated in monocytes of patients with colon but no rectal cancer. PFKFB3 is a gene that encodes the protein 6-phosphofructo-2-kinase/fructose-2,6-biphosphatase 3. This enzyme is needed to convert glucose-derived metabolite fructose-6-phosphate into fructose-2,6-biphosphate, which later is needed for glycolysis [10]. The role of PFKFB3 in the glycolytic pathway in cancer cells is well established, and has been under consideration for the therapeutic targeting in various cancer types [10,11]. However, its role in metabolic programing of TAMs is only emerging. In monocyte-derived peritumoral macrophages in hepatocellular carcinoma (HCC), PFKFB3 was found to modulate the metabolic switch and the increased NF-κB-dependent PD-L1 expression [12]. The levels of PFKFB3+CD68+ macrophage infiltration in peritumoral tissues negatively correlated with overall survival in patients with HCC. We followed the fate of PFKFB3+ monocytes in colon cancer, and using spatial transcriptomics demonstrated their massive infiltration and differentiation into PFKFB3+CD68+ [13]. PFKFB3+CD68+ correlated with unfavorable prognosis specifically in colon cancer. However, whether such elevated expression of PFKFB3 is an adaptation of macrophages to the increased energy requirements, remains unknown.

In our study we demonstrate that PFKFB3 is predominantly expressed in TAMs in CRC tumor microenvironment. For the first time by using mass spectrometry, we identified that inhibition of glycolysis-activating enzyme PFKFB3 re-programs lipid metabolism in primary human monocyte-derived TAMs.

## 2. Results

### 2.1. PFKFB3 Is Expressed in Tumor Microinvironment in Colorecal Cancer as Well as in Benign Neoplasms

**Figure 1:**
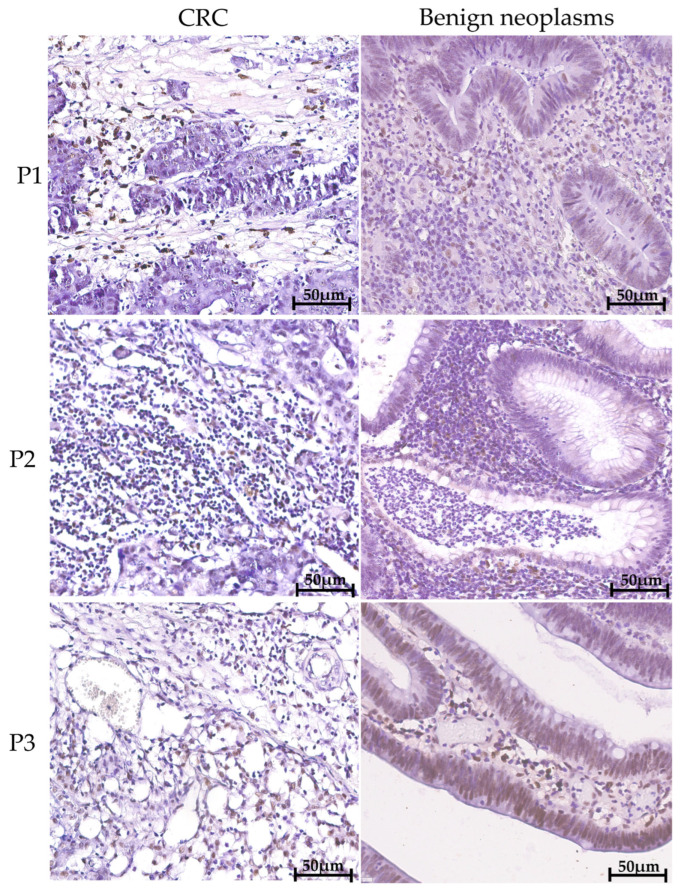
**PFKFB3+ cells are present in CRC tissue as well as in the tissue of benign neoplasms.** Immunohistochemical analysis of all samples demonstrated the presence of PFKFB3+ cells within the TME. Representative immunohistochemical images of PFKFB3 staining (brown) in tissues from benign neoplasms and colorectal cancer (CRC). Nuclei are counterstained with hematoxylin (blue). Scale bar = 50 µm. The representative images from three patients (P1–P3) are provided. Control staining and the expression in normal adjacent tissue are provided in the Supplementary Materials (Supplementary Figures S1 and S2).

Analysis of PFKFB3 protein expression was performed in malignant and benign lesions, as well as across CRC anatomical sites (Figure 1). High number of PFKFB3+ cells was observed in tumor tissues, and these cells were single infiltrating cells.

**Figure 2:**
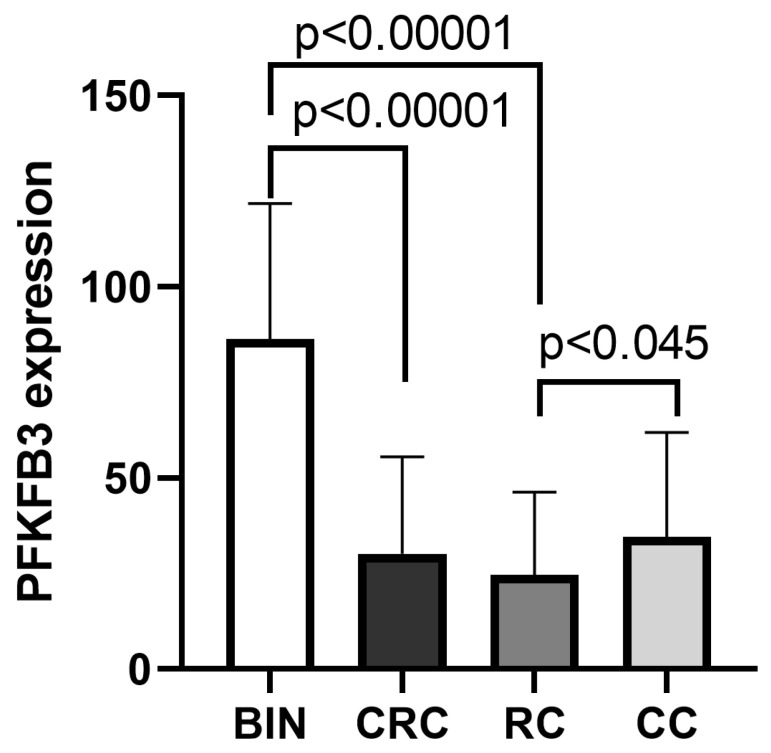
**Quantification of PFKFB3 expression in benign intestinal neoplasms and tumor tissue in colorectal cancer.** Bar graph demonstrates the mean density (with standard deviation) of PFKFB3-positive cells in the tumor stroma across patient groups: benign intestinal neoplasms (BIN), colorectal cancer (CRC), and its subtypes—colon cancer (CC) and rectal cancer (RC). Statistical significance was determined using the Mann–Whitney U test. Group comparisons are indicated by brackets. Benign neoplasms exhibit significantly higher stromal PFKFB3 expression compared to malignant CRC tissues. Within CRC, colon cancer demonstrates higher PFKFB3 levels than rectal cancer.

Notably, benign intestinal neoplasms exhibited a significantly higher density of PFKFB3^+^ cells within the stroma compared to CRC tissues (86.32 ± 35.54 vs. 30.19 ± 25.33, *p* < 0.00001). This difference was particularly pronounced when comparing benign lesions to RC (3.2-fold increase; 86.32 ± 35.54 vs. 24.72 ± 21.53, *p* < 0.00001) and to CC (2.3-fold increase; 86.32 ± 35.54 vs. 34.50 ± 27.37, *p* < 0.00001) (Table 1, Figure 2).

Within the CRC cohort, the CC subgroup maintained significantly higher PFKFB3 expression than RC (1.4-fold increase; 34.50 ± 27.37 vs. 24.72 ± 21.53, *p* < 0.045), corroborating prior monocyte data and underscoring a specific role for PFKFB3 in colon cancer pathogenesis [13]. Furthermore, tumor compartments exhibited a 3.8-fold higher PFKFB3 expression relative to matched stromal regions (65.94 ± 50.59 vs. 17.27 ± 17.50, *p* < 0.00001).

**Figure 3:**
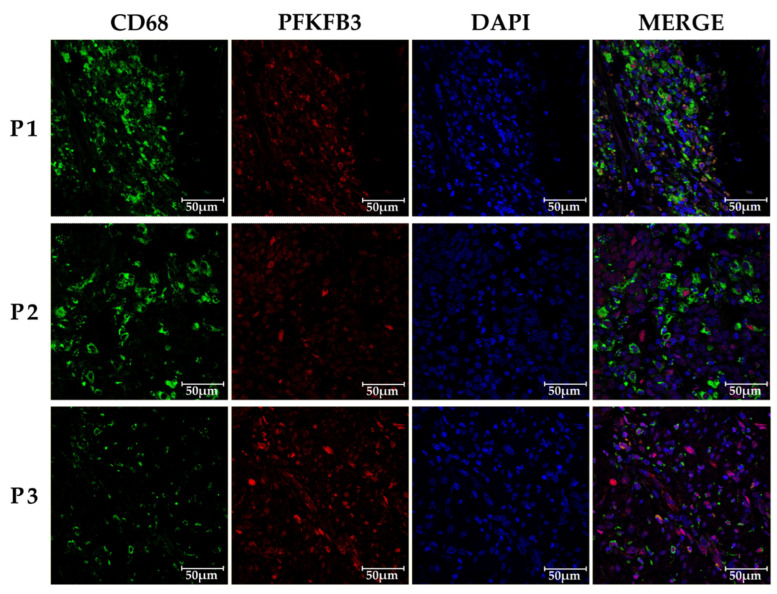
**PFKFB3 is preferentiallyexpressed in TAMs in human colon cancer tissues.** Representative confocal microscopy images of human colon cancer tissues stained with antibodies against PFKFB3 (red), the macrophage marker CD68 (green), and DAPI for nuclei (blue). The merged image shows co-localization of PFKFB3 and CD68 (orange), confirming PFKFB3 expression in TAMs. Scale bar = 50 µm. This observation emphasizes the critical role of stromal cells, particularly TAMs, in the metabolic reprogramming of the tumor niche. Our findings support the importance of investigating cell-specific contributions to PFKFB3-mediated pathways in CRC development. Specifically, the role of PFKFB3 expression in TAMs may be critical for regulating immunosuppression and metabolic adaptation within the TME.

We next analyzed whether PFKFB3 is pronominally expressed in TAMs, but not in cancer cells. Confocal microscopy analysis demonstrated TAMs is the major cell type expressing PFKFB3 (Figure 3).

### 2.2. PFKFB3 Inhibition Shifts TAM Metabolism Toward Immunosuppressive Sphingolipid and Pro-Inflammatory Arachidonic Acid Pathways

Our next question was whether inhibition of PFKFB3 can also affect other metabolic pathways in TAMs. To address this, we used an in vitro model of primary human TAMs. Monocytes were isolated from peripheral blood mononuclear cells (PBMCs) of healthy donors via CD14-positive selection and differentiated into macrophages in the presence of with M-CSF [14]. To model the tumor microenvironment, macrophages were polarized toward TAMs by conditioned medium from the human colorectal cancer cell line Caco2. PFKFB3 inhibition was then performed using two structurally distinct compounds: PFK15, a known PFKFB3 inhibitor, and AZ PFKFB3 26, a potent, selective, and ATP-competitive small-molecule inhibitor of the PFKFB3 kinase domain. Both inhibitors suppress glycolytic flux by decreasing intracellular levels of fructose-2,6-bisphosphate, the key allosteric activator of phosphofructokinase-1. Using MetaboAnalyst and the KEGG database, we mapped these lipid changes to biological pathways, ranking them by both statistical significance and overall metabolic impact (Table 2 and Table 3).

**Figure 4:**
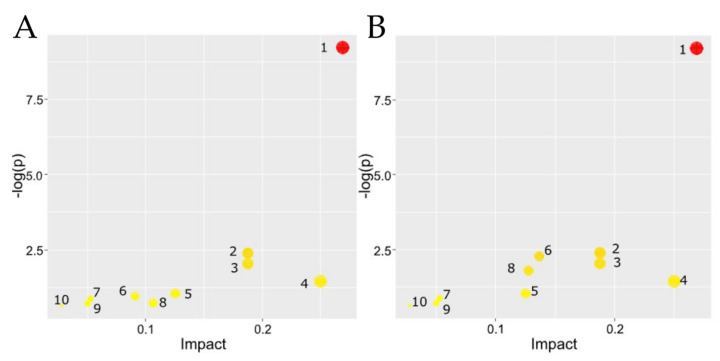
Pathway analysis of lipidomic changes in primary human monocyte-derived model TAMs following PFKFB3 inhibition with PFK15 and AZ PFKFB3 26. Both inhibitors most profoundly affect glycerophospholipid metabolism but exhibit distinct secondary effects, with PFK15 showing a stronger association with ether lipid metabolism and AZ PFKFB3 26 having a greater impact on the phosphatidylinositol signaling system. Pathway analysis plots based on data from Table 3 and Table 4. (**A**) Control vs. PFK15 treatment. (**B**) Control vs. AZ PFKFB3 26 treatment. Each node represents a metabolic pathway. Node color corresponds to the level of statistical significance (−log10(*p*-value)), from red (most significant) to yellow (less significant). Node radius corresponds to the “Pathway Impact” value, which reflects the cumulative influence of the altered metabolites on the pathway as a whole. Numbered pathways: glycerophospholipid metabolism (1); glycosylphosphatidylinositol (GPI)-anchor biosynthesis (2); sphingolipid metabolism (3); linoleic acid metabolism (4); alpha-linolenic acid metabolism (5); glycerolipid metabolism (6); ether lipid metabolism (7); phosphatidylinositol signaling system (8); inositol phosphate metabolism (9) and arachidonic acid metabolism (10).

This approach revealed that PFKFB3 inhibition profoundly redirects TAM metabolism, particularly toward immunosuppressive sphingolipid and pro-inflammatory arachidonic acid pathways. The following section presents a detailed analysis of these metabolic pathways and their potential implications for the TME (Figure 4).

**Figure 5:**
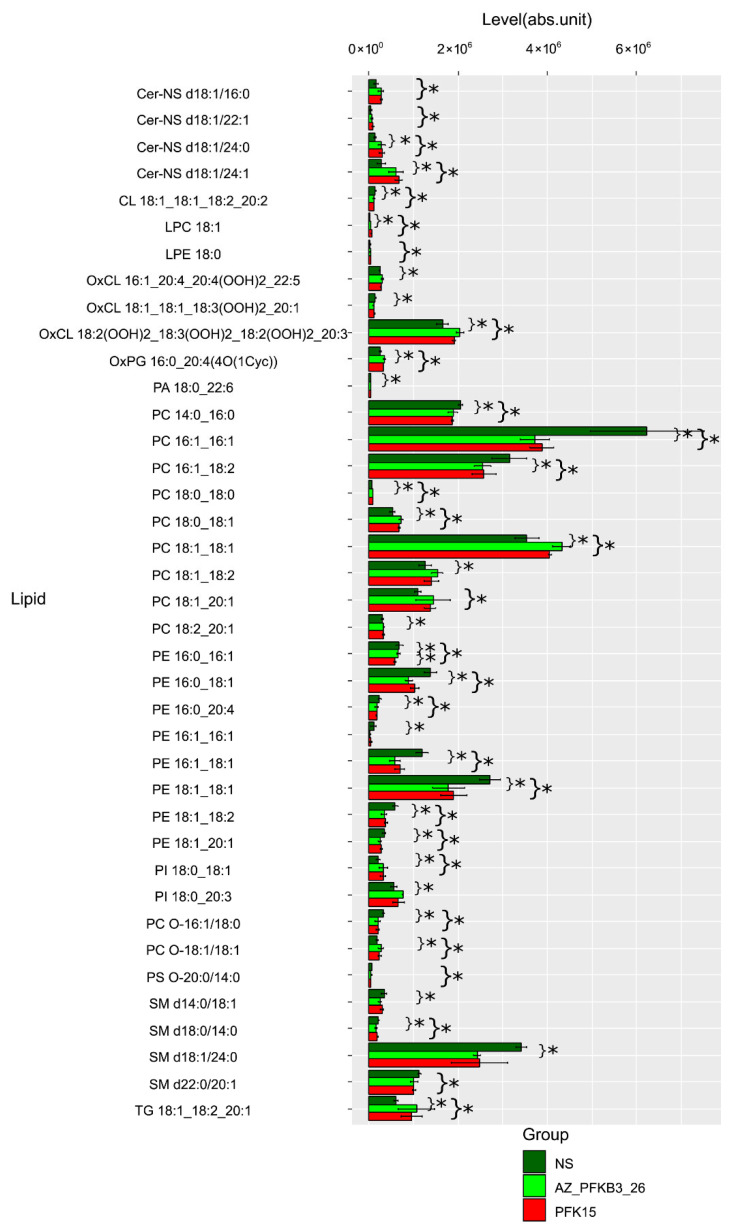
Lipidomic profiles of primary human monocyte-derive model TAMs following PFKFB3 inhibition with PFK15 and AZ PFKFB3 26. The profiles confirm significant alterations in key lipid species, including a decrease in glycerophospholipids (PC, PE) and an increase in specific ceramides and oxidized lipids upon PFKFB3 inhibition, supporting the pathway analysis findings. The panels show levels of individual lipids, grouped by class, that demonstrated statistically significant differences in pairwise comparisons between non-treated control (NS), PFK15-treated, and AZ PFKFB3 26-treated groups (pairwise Mann–Whitney U tests). Data are presented as median values with standard deviation. Asterisks denote statistical significance (* *p* < 0.05) compared to the control group, unless otherwise indicated. Lipid classes: Cer—ceramides, PC—phosphatidylcholines, PE—phosphatidylethanolamines, OxPL—oxidized phospholipids, PS—phosphatidylserines, LPC—lysophosphatidylcholines, CL—cardiolipins.

Two hundred lipids were identified in both positive and negative ion modes, primarily belonging to triglyceride, ceramide, sphingomyelin, cardiolipin, fatty acid, phosphatidylinositol, various phosphatidylcholine and phosphatidylethanolamine classes, including their oxidized derivatives. Figure 5 presents comparative lipid levels across macrophage groups.

Lipid metabolism potentially influences the pro- or anti-inflammatory activities of macrophages by fulfilling their energy demands and regulating membrane fluidity (Table 4).

Various lipids, including free fatty acids (FFAs), neutral lipids, glycerophospholipids (GLs), and sphingolipid, constitute cell membranes and lipoproteins, as well as play critical roles in diverse biological processes such as inflammation and cell differentiation [23]. Pathway enrichment analysis using KEGG and MetaboAnalyst highlighted glycerophospholipid metabolism as the most significantly affected pathway (*p* = 6.13 × 10^−10^) in both inhibitor-treated groups, with notable involvement of glycosylphosphatidylinositol (GPI)-anchor biosynthesis, sphingolipid metabolism, and linoleic acid metabolism. The impact of PFK15 and AZ PFKFB3 26 diverged in specific pathways: PFK15 exhibited stronger associations with ether lipid metabolism and arachidonic acid metabolism, while PFKB3_26 showed greater influence on glycerolipid metabolism and phosphatidylinositol signaling. These findings suggest that both inhibitors disrupt membrane lipid homeostasis and inflammatory signaling networks.

The identified lipidomic shifts imply that inhibition of PFKFB3 in TAMs alters metabolic fluxes, particularly in pathways linked to pro-inflammatory (e.g., arachidonic acid derivatives) and immunosuppressive (e.g., sphingolipids) mediators. The consistent downregulation of glycerophospholipids, critical for membrane structure and signaling, may impair macrophage functionality in the TME. Furthermore, the differential effects on linoleic and alpha-linolenic acid metabolism suggest context-dependent roles for these lipids in CRC progression.

### 2.3. PFKFB3 Inhibition Affects Amino Acid Metabolism in Macrophages

To further characterize the systemic metabolic impact of PFKFB3 inhibition in our primary human TAM model, we extended our analysis to the amino acid repertoire in macrophages. Utilizing high-performance liquid chromatography coupled with tandem mass spectrometry in multiple reaction monitoring mode (HPLC-MS/MS-MRM), we quantitatively profiled the levels of 20 amino acids.

Notably, control samples occupied an intermediate position in the principal component space, situated between the two separate clusters formed by PFK15 and AZ PFKFB3 26-treated groups. This clear separation indicates that PFKFB3 inhibition induces a profound and specific reprogramming of amino acid metabolism, suggesting a metabolic rewiring that extends beyond carbon flux through glycolysis to encompass nitrogen metabolism and protein synthesis precursors.

**Figure 6:**
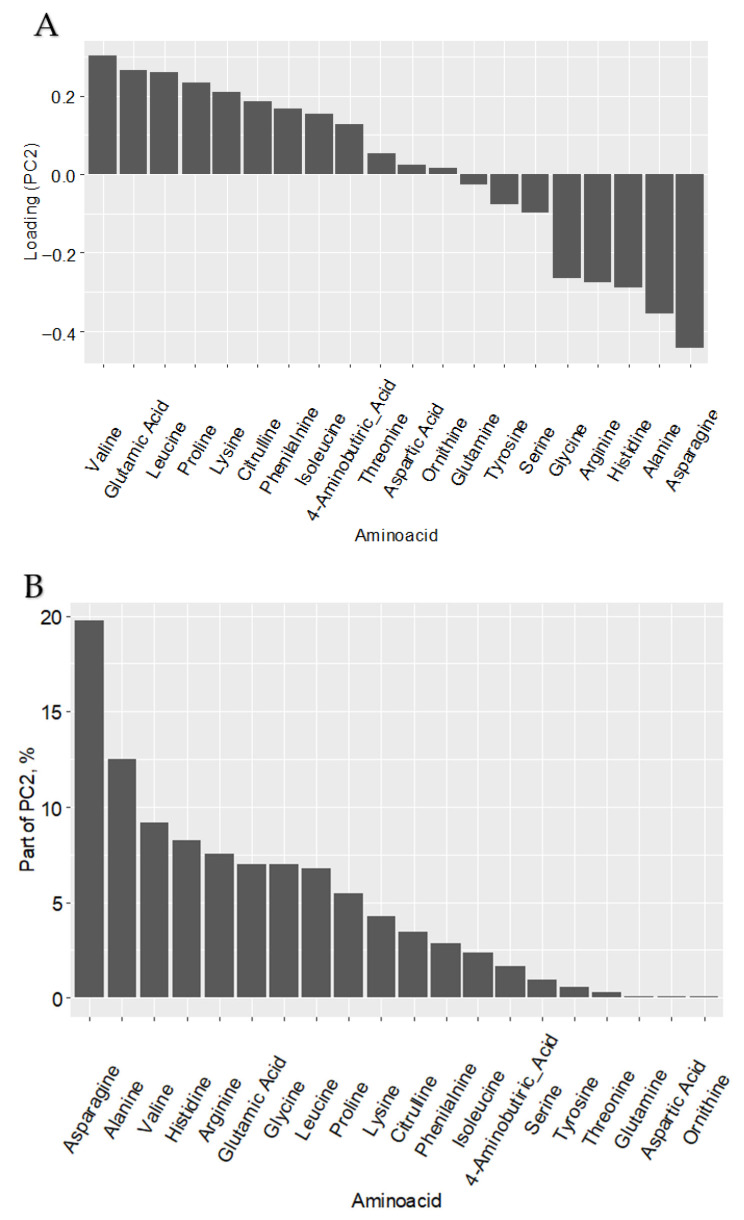
PFKFB3 inhibition reprograms the amino acid metabolism of tumor-associated macrophages. The distinct clustering of PFKFB3-inhibited macrophages is primarily driven by a coordinated reduction in asparagine levels, highlighting its role as a key metabolic node in the amino acid reprogramming of TAMs. This indicates that modulation of PFKFB3 activity is associated with a specific reduction in asparagine availability, potentially pointing to a targeted reprogramming of amino acid metabolism that may support distinct macrophage functions under conditions of metabolic stress. (**A**) Principal Component Analysis (PCA) of amino acid profiles from control (NS), PFK15-treated, and AZ PFKFB3 26-treated macrophages. Each point represents an individual sample. (**B**) Analysis of variable importance for PC2. Upper bar chart shows loadings of each amino acid on PC2. Lower bar chart shows percentage contribution of each amino acid to PC2 variance. Asparagine demonstrates both the strongest negative loading and the largest contribution to PC2.

This observed clustering, despite the absence of statistically significant differences in individual amino acids by Mann–Whitney U test, suggests subtle but coordinated shifts in the amino acid profile that are associated with inhibitor treatment. The clear separation of groups along the second principal component (PC2) prompted an investigation into the variables driving this distribution. Variable loading analysis (Figure 6) identified asparagine as the dominant contributor to the separation along PC2, with its levels decreasing progressively along this axis in the inhibitor-treated groups.

## 3. Discussion

This study provides compelling evidence that CRC cells actively orchestrate the metabolic reprogramming of TAMs by inducing the expression of the glycolytic regulator PFKFB3. Our findings reveal a complex interplay between tumor-derived signals and macrophage metabolism, which culminates in the promotion of a pro-tumoral phenotype of TAMs characterized by a distinct lipidomic profile. The significantly higher expression of PFKFB3 in the stroma of benign neoplasms compared to malignant CRC tissues suggests a dynamic regulation of this metabolic enzyme during cancer progression, potentially reflecting an early host anti-tumor response that becomes suppressed or reprogrammed in advanced disease. Our observation that colon cancer tissues exhibit higher PFKFB3 expression than rectal cancer tissues further underscore the metabolic heterogeneity within CRC subtypes and aligns with our previous findings of elevated PFKFB3 in monocytes of colon cancer patients, pointing to a subtype-specific role for this metabolic pathway in disease pathogenesis [13]. This aligns with the emerging concept that cancer therapy can systemically reprogram monocyte precursors and their differentiation, influencing the metabolic and functional polarization of TAMs [24]. These findings comply with our previous recent study that established prognostic role of PFKFB3 in colon cancer, but not in rectal cancer. In colon cancer, PFKFB3 overexpression correlated with a higher risk of tumor relapse and poor survival rates [13].

Our comprehensive metabolic analysis demonstrates that PFKFB3 inhibition induces profound and multifaceted reprogramming of TAM metabolism, extending beyond glycolytic suppression to encompass significant alterations in lipid and amino acid metabolic pathways. The observed shifts reveal a paradoxical dual effect: while effectively reducing monocyte recruitment and suppressing certain pro-inflammatory mediators, PFKFB3 inhibition simultaneously promotes immunosuppressive metabolic reprograming. Specifically, both inhibitors consistently disrupted glycerophospholipid metabolism, critical for membrane integrity, while differentially modulating sphingolipid and inflammatory pathways.

Most intriguing is the lipidomic changes in macrophages after PFKFB3 inhibition, which revealed a profound metabolic plasticity in TAMs. Significant alteration in glycerophospholipid metabolism suggests substantial membrane remodeling, which could impact numerous cellular functions including phagocytosis, cytokine secretion, cell signaling, in M2 macrophage polarization and anti-inflammatory processes [25,26]. The shift toward sphingolipid metabolism is particularly noteworthy given the established role of sphingolipids in promoting immunosuppressive and M2-like macrophage polarization [26,27]. Simultaneously, the observed changes in linoleic and arachidonic acid metabolism indicate modulation of inflammatory signaling pathways, creating a complex metabolic landscape where PFKFB3 inhibition may simultaneously impair some pro-tumoral functions while potentially reinforcing others through alternative metabolic routes. This metabolic flexibility may represent an adaptive mechanism that allows TAMs to maintain immunosuppressive functions even under glycolytic restriction, explaining why PFKFB3 inhibition alone may be insufficient to completely reverse the immunosuppressive TME.

Notably, the core metabolic phenotypes—particularly the profound disruption of glycerophospholipid metabolism and the shift toward sphingolipid synthesis—were consistently observed after the application of two chemically distinct PFKFB3 inhibitors, PFK15 and AZ PFKFB3 26. This convergence of findings strongly suggests that the observed reprogramming is a direct consequence of PFKFB3 blockade itself, rather than an off-target artifact unique to either compound. The subtle differences in their impact on specific pathways (e.g., the stronger association of PFK15 with ether lipid metabolism and of AZ PFKFB3 26 with phosphatidylinositol signaling) may reflect variations in their pharmacodynamic profiles or secondary targets, yet they collectively emphasize the central role of PFKFB3 as a metabolic node governing TAMs polarization.

The co-occurrence of sphingolipid enrichment and alterations in arachidonic acid (AA) metabolism following PFKFB3 inhibition, while seemingly paradoxical, likely reflects a complex, integrated metabolic adaptation rather than a simple mix of pro- and anti-inflammatory states. Firstly, the traditional view of AA metabolites as universally ‘pro-inflammatory’ is an oversimplification. In the tumor microenvironment, specific AA-derived mediators, such as prostaglandin E2 (PGE2), are well-established drivers of immunosuppression, contributing to TAMs polarization, T-cell inhibition, and angiogenesis [28,29,30]. Thus, their modulation may support the overall immunosuppressive landscape. Secondly, the concurrent rise in specific ceramides and oxidized phospholipids points towards a state of metabolic stress triggered by glycolytic restriction. Sphingolipids are key mediators of stress responses and can directly inhibit pro-inflammatory signaling (e.g., via modulation of TLR pathways), promoting an M2-like, survival-oriented phenotype [31,32,33]. The generation of oxidized lipids, potentially stemming from enhanced mitochondrial activity or redox imbalance under metabolic stress, may serve as signaling molecules that fine-tune this adaptation rather than eliciting a robust classical inflammatory response [9]. Therefore, the lipidomic signature we describe is not contradictory but likely reflects a specific metabolic state or configuration of TAMs that enables the maintenance of pro-tumoral functions under the constraint of PFKFB3 inhibition. This highlights the remarkable metabolic plasticity of TAMs and suggests that targeting PFKFB3 alone may be insufficient to reverse their phenotype, as it triggers compensatory rewiring toward alternative immunosuppressive pathways.

The distinct clustering patterns in amino acid profiles following PFKFB3 inhibition, driven primarily by reduced asparagine availability, further expanded our understanding of the metabolic reprograming beyond carbon metabolism to include nitrogen metabolism. The distinct clustering patterns in amino acid profiles, driven primarily by reduced asparagine availability, further indicate that PFKFB3 blockade triggers coordinated nitrogen metabolic reprograming that may support macrophage adaptation to metabolic stress. This coordinated reprogramming suggests that PFKFB3 serves as a metabolic integrator coordinated multiple metabolic pathways to support tumor-promoting functions of TAMs.

## 4. Materials and Methods

### 4.1. Patients

The study enrolled patients (*n* = 111) with morphologically confirmed colorectal adenocarcinoma treated at the Department of Abdominal Oncology, Cancer Research Institute of Tomsk National Research Medical Center (Tomsk, Russia) between 2019 and 2021, along with healthy donors. Conducted in accordance with the Declaration of Helsinki (1964, revised 1975 and 1983), the study was approved by the Local Ethics Committee of the Tomsk Cancer Research Institute (protocol number 7 dated 25 August 2020), with all participants providing written informed consent. Patients were stratified into two groups based on tumor type and treatment approach: colon cancer patients (T2-4N0-3M0, stages I–III) and rectal cancer patients (T2-4N0-3M0, stages I–III). Colon cancers involved various segments, including the cecum, ascending, transverse, descending, and sigmoid colon.

Rectal cancer patients underwent surgery, followed by adjuvant chemotherapy (using the same regimens) for up to six months. Rectal cancers included tumors of the rectum and rectosigmoid junction.

For comparative immunohistochemical analysis of the tumor microenvironment, a control group of patients with benign intestinal neoplasms (*n* = 50) was additionally enrolled. This group consisted of individuals with histologically verified benign lesions, including tubular adenomas, tubulovillous adenomas, and hyperplastic polyps, which were identified and resected during standard diagnostic or screening colonoscopies. Patients with benign neoplasms were selected to provide a representative baseline for stromal and immune cell characteristics in non-malignant intestinal tissue, allowing for a direct comparison with the malignant cohorts.

Healthy volunteers, matched for age and sex, served as controls. Eligibility criteria for healthy participants included age 48–70 years, absence of acute inflammatory or severe chronic conditions (e.g., diabetes, hepatitis, HIV, myocarditis), no immunomodulatory drug use within 30 days prior to enrollment, capacity to provide informed consent, and no personal history of malignancy.

### 4.2. Immunohistochemical (IHC) Analysis

Quantitative assessment of PFKFB3-positive TAMs in TME and benign intestinal tumors (*n* = 50) was performed. Standard IHC staining procedures were employed on FFPE tissue sections using the following primary antibodies: rabbit monoclonal anti-PFKFB3 (Abcam, Cambridge, UK ab181861, dilution 1:50), mouse monoclonal anti-CD68 (Novus Biologicals, Colorado, USA, NBP2 445-39, clone KP1, dilution 1:100). For mouse and rabbit antibodies, the Bond Oracle IHC system (TA9145, Leica Biosystems, Deerfield, IL, USA) was used for visualization with DAB chromogen. Nuclear counterstaining was performed with hematoxylin. Tumor tissue sections were scanned using a Leica Aperio AT2 slide scanner (Deerfield, IL, USA) and ScanScope software (IL, Deerfield, USA). QuPath software version 0.5.2 (Edinburgh, UK) (freely available from https://qupath.github.io (accessed on 3 November 2024)) was utilized for image analysis and marker quantification. Regions of interest were selected and analyzed using cell detection and intensity classification. “Cell: Mean DAB OD” was employed to analyze both membrane and cytoplasmic staining. Intensity thresholds were set to further classify cells as negative, weak, moderate, or strong for PFKFB3 staining based on the mean nuclear DAB optical density.

### 4.3. Immunofluorescent Staining and Confocal Microscopy

FFPE tissue sections from ten colon cancer patients were utilized for immunohistochemical analysis. Antigen retrieval was performed using the PT Link module (Dako, Glostrup, Denmark) in T/E buffer (pH 9.0). For immunofluorescence (IF) staining, FFPE tumor sections were deparaffinized in xylol, and subsequently blocked with 3% BSA in PBS for 45 min. Sections were then incubated with primary antibodies for 1.5 h, washed, and incubated with appropriate secondary antibodies for 45 min. The following primary antibodies were used: rabbit monoclonal anti-PFKFB3 (1:50, #ab181861, Abcam, Cambridge, UK,), mouse monoclonal anti-CD68 (1:100, clone KP1, #NBP2-44539, Novus Biologicals). Secondary antibodies included donkey Cy3-conjugated anti-rabbit antibody (1:400, #711-165-152, Dianova, Hamburg, Germany), donkey AlexaFluor488-conjugated anti-mouse antibody (1:400, #715-545-150, Dianova, Germany), and slides were mounted with Fluoroshield Mounting Medium with DAPI (#ab104135, Abcam, Cambridge, UK). Confocal microscopy was performed using a Carl Zeiss LSM 780 NLO laser scanning spectral confocal microscope (Carl Zeiss, Jena, Germany) equipped with a 40x objective. Images were acquired and analyzed using ZEN software (Jena, Germany) (RRID: SCR_018163) in sequential scan mode for all four-color images.

### 4.4. Isolation of Monocytes and Model of Primary Human TAMs

Monocytes were isolated from human buffy coats using magnetic-activated cell sorting (MACS) with CD14-positive selection as previously described [14]. The resulting cell population contained 95–98% monocytes, as confirmed by flow cytometry analysis of CD14 surface expression. Monocytes from four healthy donors were cultured at a concentration of 2 × 10^6^ cells/mL at 37 °C for 6 days in X-VIVO medium. All macrophages were stimulated with M-CSF (1 ng/mL) and dexamethasone (10^−8^ M). For M1 polarization, IFNγ (100 ng/mL) was added; for M2 polarization, IL-4 (10 ng/mL) was added; and for tumor-associated macrophage (TAM) differentiation, 80% X-VIVO and 20% tumor supernatant from the colorectal cancer cell line Caco2 were used. PFKFB3 inhibition was performed using PFK15 (207 nM), a known PFKFB3 inhibitor, and AZ PFKFB3 26 (0.023 μM), a potent and selective ATP-competitive small-molecule inhibitor of the PFKFB3 kinase domain (Tocris, Bristol, UK) after monocyte-to macrophage differentiation. After 6 days of culture, cells were harvested and used for HPLC-MS/MS (Agilent, Santa Clara, CA, USA) analysis.

### 4.5. Macrophage Lipidome Analysis

The macrophage lipidome was characterized using HPLC-MS/MS analysis of lipid extracts. Monocyte samples were mixed with 480 μL of chloroform-methanol (2:1, *v*/*v*) at 4 °C, followed by 10 min sonication. After adding 150 μL of water, the solution was centrifuged at 13,000× *g* for 5 min at room temperature. The lower organic layer containing lipids was collected, dried under nitrogen stream, and reconstituted in 200 μL of isopropanol-acetonitrile (1:1, *v*/*v*). Lipid extracts were analyzed using a Dionex UltiMate 3000 liquid chromatograph (Thermo Scientific, Waltham, MA, USA) coupled to a Maxis Impact qTOF mass spectrometer with an ESI ion source (Bruker Daltonics, Bremen, Germany). Reverse-phase chromatography was performed on a Zorbax C18 column (150 × 2.1 mm, 5 μM; Agilent, Santa Clara, CA, USA) with a 20 min linear gradient from 30% to 90% eluent B (acetonitrile/isopropanol/water, 90/8/2 *v*/*v*/*v*, containing 0.1% formic acid and 10 mmol/L ammonium formate). Eluent A consisted of acetonitrile/water (60/40, *v*/*v*) with 0.1% formic acid and 10 mmol/L ammonium formate. The flow rate was maintained at 35 μL/min with a 1 μL injection volume. Mass spectra were acquired in positive and negative ion modes (*m*/*z* 100–1700) with the following parameters: capillary voltage 4.1 kV, nebulizer gas pressure 0.7 bar, drying gas flow rate 6 L/min, and drying gas temperature 200 °C. Lipid identification was performed using tandem mass spectrometry in data-dependent acquisition mode with a 5 Da isolation window.

Chromatographic data were preprocessed using Proteowizard 3.0.9987 (msConvert) to generate MzXML (full scan) and MS2 (tandem spectra) files, followed by peak detection and feature table generation in MZmine 2.20. Lipids were identified using LipidMatch [26] with a mass accuracy of 0.01 Da, fragment ion matching within 5 Da mass windows, and retention time tolerances of 0.5 min. Statistical analysis was performed using custom R scripts in RStudio 2023.09.1 Build 494 (Boston, MA, USA), comparing lipid levels between control macrophages and those treated with PFK15 or AZ PFKFB3 26 (pairwise comparisons, *p* < 0.05). Principal component analysis (PCA) was employed to assess global lipid profile differences.

### 4.6. Amino Acid Profile Analysis

The amino acid profile analysis was performed using HPLC-MS/MS on a system comprising an Agilent 6460 triple quadrupole mass spectrometer (Agilent, Santa Clara, CA, USA) equipped with an electrospray ionization source coupled to an Agilent 1260 II liquid chromatograph with a binary high-pressure pump, column thermostat, and 122-vial autosampler. Sample preparation followed this protocol: macrophage samples were mixed with 480 μL of chloroform-methanol (2:1, *v*/*v*) at 4 °C, sonicated for 10 min, then supplemented with 150 μL of water and vortexed for 5 min. After centrifugation at 13,000× *g* for 5 min at room temperature, 150 μL of the upper aqueous-methanolic layer was collected, evaporated under nitrogen stream for 30 min at 60 °C, and reconstituted in 200 μL of 0.1 M hydrochloric acid in butanol. The mixture was vortexed for 3 min, centrifuged at 13,000× *g* for 15 s, then incubated at 60 °C for 15 min to complete derivatization. Following another centrifugation (13,000× *g*, 15 s), samples were dried under nitrogen (30 min, 60 °C), redissolved in 300 μL of acetonitrile/water (1:1, *v*/*v*), vortexed for 5 min, and centrifuged (13,000× *g*, 10 min). Finally, 200 μL aliquots were transferred to vials with inserts for analysis.

Statistical analysis was performed using custom R scripts in RStudio. Amino acid levels in macrophages from colorectal cancer patients were compared pairwise between control, PFK15-treated, and PFKB3_26-treated groups using Student’s *t*-test with a significance threshold of *p* < 0.05. Principal component analysis was employed to evaluate global differences in amino acid profiles.

### 4.7. Statistical Analysis

Statistical analysis was performed using STATISTICA 8.0 for Windows (STATISTICA (Palo Alto, CA, USA), RRID: SCR_014213) and GraphPad Prism 8.4.2 (GraphPad Prism, RRID: SCR_002798). The Manna-Whitney test and *t*-test for independent groups were implemented.

## 5. Conclusions

In conclusion, our study demonstrates that CRC cells strongly induce PFKFB3 expression in TAMs, thereby promoting their metabolic and functional reprogramming. We found that the stromal infiltration of PFKFB3^+^ cells is significantly elevated in colorectal tumors, particularly in colon cancer, and is associated with increased infiltration of pro-tumoral macrophages. Immunofluorescence analysis identified that PFKFB3 expressed predominantly in CD68^+^ TAMs. Lipidomic profiling revealed that PFKFB3 inhibition profoundly alters glycerophospholipid metabolism and shifts macrophage lipidomes toward immunosuppressive sphingolipid and pro-inflammatory arachidonic acid pathways. Additionally, amino acid metabolic profiling indicated coordinated changes in nitrogen metabolism, particularly a reduction in asparagine availability. These findings establish PFKFB3 as a central metabolic regulator in TAMs, governing both their recruitment and pro-tumoral polarization within the colorectal tumor microenvironment. Targeting PFKFB3 may therefore represent a promising therapeutic strategy to disrupt tumor–macrophage crosstalk and impede cancer progression, although the metabolic plasticity of TAMs suggests that combination therapies addressing multiple pathways may be necessary to fully overcome tumor-induced immunosuppression.

## Figures and Tables

**Table 1:** Comparative analysis of stromal PFKFB3^+^ cell density in the tumor stroma of patients with colorectal cancer and benign intestinal neoplasms. Data represent the density of all PFKFB3-positive cells within the stromal compartment. The stroma contains a heterogeneous mix of cell types (e.g., fibroblasts, immune cells); therefore, this quantification does not specify the cellular source of PFKFB3. For cell-type-specific analysis, see Figure 3 (co-localization with CD68^+^ macrophages).

Benign Intestinal Neoplasms (1)	Colorectal Cancer (2)	Rectal Cancer (3)	Colon Cancer (4)
86.32 ± 35.54 * (*n* = 50) *p*_1,2_ < 0.00001	30.19 ± 25.33 (*n* = 111)	24.72 ± 21.53 (*n* = 49) *p*_1,3_ < 0.00001 *p*_3,4_ < 0.045	34.50 ± 27.37 (*n* = 62) *p*_3,4_ < 0.045

* Data are presented as mean ± standard deviation of PFKFB3+ cell counts per field of view. Statistical significance between groups was determined using Student’s *t*-test. Group comparisons: (1) benign intestinal neoplasms (BIN), (2) All CRC cases, (3) rectal cancer (RC), (4) colon cancer (CC). *p*-values are denoted as *p*_1,2_~ (comparison of groups (1) and (2)), etc.

**Table 2:** Metabolic pathway enrichment analysis of lipids altered in PFK15-treated macrophages compared to control.

Name Metabolic Pathways	The Total Number of Metabolites Involved	The Number of Metabolites Matching the Experimental Data	*p*	LOG(*p*)	Effect on Pathway
Glycerophospholipid metabolism	36	7	6.13 × 10^−10^	9.2127	0.26922
Glycosylphosphatidylinositol (GPI)-anchor biosynthesis	14	2	0.003979	2.4002	0.1875
Sphingolipid metabolism	21	2	0.008937	2.0488	0.1875
Linoleic acid metabolism	5	1	0.035028	1.4556	0.25
alpha-Linolenic acid metabolism	13	1	0.08876	1.0518	0.125
Glycerolipid metabolism	16	1	0.1082	0.96578	0.09091
Ether lipid metabolism	20	1	0.13353	0.87443	0.05263
Phosphatidylinositol signaling system	28	1	0.18224	0.73936	0.10638
Inositol phosphate metabolism	30	1	0.19402	0.71216	0.05
Arachidonic acid metabolism	36	1	0.22844	0.64122	0.02778

Pathway enrichment analysis was performed using MetaboAnalyst and the KEGG database for lipids that exhibited statistically significant alterations following PFK15 treatment. “Total” is the total number of metabolites in the pathway according to KEGG; “Hits” is the number of metabolites from our dataset matching the pathway; “*p*” is the *p*-value from the enrichment analysis; “Effect on pathway” is the calculated pathway impact value. Pathways are ranked by *p*-value.

**Table 3:** Metabolic pathway enrichment analysis of lipids altered in AZ PFKFB3 26-treated macrophages compared to control.

Name Metabolic Pathways	The Total Number of Metabolites Involved	The Number of Metabolites Matching the Experimental Data	*p*	−LOG(*p*)	Effect on Pathway
Glycerophospholipid metabolism	36	7	6.13 × 10^−10^	9.2127	0.26922
Glycosylphosphatidylinositol (GPI)-anchor biosynthesis	14	2	0.003979	2.4002	0.1875
Glycerolipid metabolism	16	2	0.005207	2.2834	0.13636
Sphingolipid metabolism	21	2	0.008937	2.0488	0.1875
Phosphatidylinositol signaling system	28	2	0.015655	1.8053	0.12766
Linoleic acid metabolism	5	1	0.035028	1.4556	0.25
alpha-Linolenic acid metabolism	13	1	0.08876	1.0518	0.125
Ether lipid metabolism	20	1	0.13353	0.87443	0.05263
Inositol phosphate metabolism	30	1	0.19402	0.71216	0.05
Arachidonic acid metabolism	36	1	0.22844	0.64122	0.02778

Pathway enrichment analysis was performed using MetaboAnalyst and the KEGG database for lipids that exhibited statistically significant alterations following AZ PFKFB3 26 treatment. “Total” is the total number of metabolites in the pathway according to KEGG; “Hits” is the number of metabolites from our dataset matching the pathway; “*p*” is the *p*-value from the enrichment analysis; “Effect on pathway” is the calculated pathway impact value. Pathways are ranked by *p*-value.

**Table 4:** Functional consequences of PFKFB3 inhibition on macrophage lipid metabolism and polarization in colorectal cancer. Summary of the effects of PFKFB3 inhibitors on specific lipid classes and the functional consequences for TAM polarization and activity. PFKFB3 inhibition induces a complex lipid metabolic rewiring that has a dual effect: while impairing some pro-inflammatory functions, it may simultaneously promote an immunosuppressive (M2-like) phenotype through the accumulation of sphingolipids.

Inhibitor	Lipid Class	Specific Lipi ds	Regulation *	Pathway	Effects of PFKFB3 Inhibitors on TAMs
PFK15	Glycerophospholipids	Phosphatidylcholines, PCs (PC 14:0_16:0, PC 16:1_16:1, PC 16:1_18:2, PC 18:0_18:0, PC 18:0_18:1, PC 18:1_18:1, PC 18:1_18:2, PC 18:1_20:1, PC 18:2_20:1, PC O-16:1/18:0, PC O-18:1/18:1)	↓	Glycerophospholipid metabolism	Membrane destabilization Impairing of phagocytosis, monocyte migration and production of pro-inflammatory citokine production (tumor-suppressive) [15]
	Sphingolipids	Ceramides, Cer (Cer-NS d18:1/16:0, Cer-NS d18:1/22:1, Cer-NS d18:1/24:0, Cer-NS d18:1/24:1)	↑	Sphingolipid metabolism	Anti-inflammatory skewing M2-like polarization (tumor-promotive) [16]
	Fatty Acids	Linoleic acid derivatives, PCs (LPC 18:2, PC 16:1_18:2, PC 18:1_18:2, PC 18:2_20:1, PE 18:1_18:2, OxCL 18:1_18:1_18:3(OOH)2_20:1, OxCL 18:2(OOH)2_18:3(OOH)2_18:2(OOH)2_20:3)	↑	Linoleic acid metabolism	Increasing of pro-inflammatory mediators [17,18]
	Ether Lipids	Plasmalogens (PC O-16:1/18:0, PC O-18:1/18:1, PS O-20:0/14:0)	↓	Ether lipid metabolism	Diminished antioxidant capacity [19]
AZ PFKFB3 26	Glycerophospholipids	Phosphatidylethanolamines (PE 16:0_16:1, PE 16:0_18:1, PE 16:0_20:4, PE 16:1_16:1, PE 16:1_18:1, PE 18:1_18:1, PE 18:1_18:2, PE 18:1_20:1)	↓	Glycerophospholipid metabolism	Supression of M1 activation Tumor-promotive effects [20]
	Phosphoinositides	PIP2, PIP3	↓	PI signaling system of PI3K/AKT signaling pathway	Reduction in inflammatory responses [21]
	Oxidized Lipids	Oxidized phosphatidylcholines (OxPC 16:0_20:4(4O(1Oye)), OxPC 18:1_18:3(OOH)2_20:1, OxPC 18:2(OOH)2_18:3(OOH)2_18:2(OOH)2_20:3)	↑	Arachidonic acid metabolism	Suppression of lipid peroxidation and reduction in pro-inflammatory mediators, which may lead to attenuation of oxidative stress and modulation of the inflammatory response [22].

* Arrows (↑↓) in the “Regulation” column indicate an increase or decrease in lipid levels upon inhibitor treatment. References supporting the functional effects are provided in square brackets.

## Data Availability

The original contributions presented in this study are included in the article/Supplementary Material. Further inquiries can be directed to the corresponding author(s).

## Supplementary Materials

The following supporting information can be downloaded at https://www.mdpi.com/article/10.3390/ijms27010217/s1.
