# Supplementary material for: Inhibition of PFKFB3 in Macrophages Has a Dual Effect on Tumor-Regulating Lipid Metabolism
Source: Int J Mol Sci. 2025 Dec 24;27(1):217. doi: 10.3390/ijms27010217 (PMC12785469; doi:10.3390/ijms27010217)
Supplement: Supplementary file 1 [file ijms-27-00217-s001.zip › ijms-3943734-supplementary.pdf]

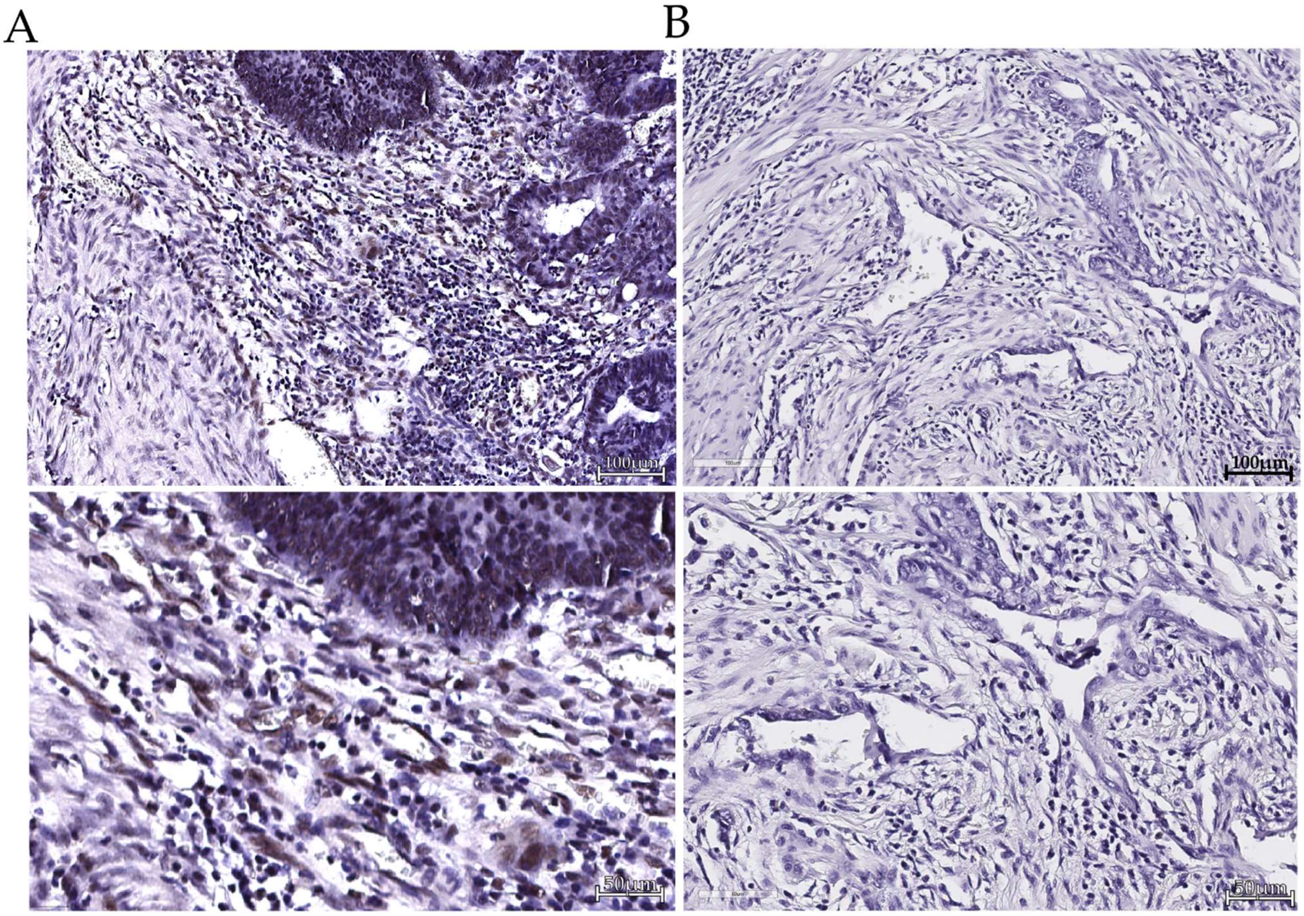

Figure S1. Rabbit polyclonal antibody control staining confirms the specificity of PFKFB3 immunohistochemistry. Representative images of colorectal cancer tissue sections stained with **(A)** the specific rabbit monoclonal anti-PFKFB3 antibody (clone ab181861, 1:50 dilution) and **(B)** a non-specific rabbit polyclonal antibody (1:50 dilution, Thermo Fisher Scientific, USA) under identical IHC conditions. Nuclear counterstaining with hematoxylin (blue). The absence of specific brown DAB staining in the isotype control validates the specificity of the signal observed with the anti-PFKFB3 antibody. Scale bar = 100 μm and 50 μm.

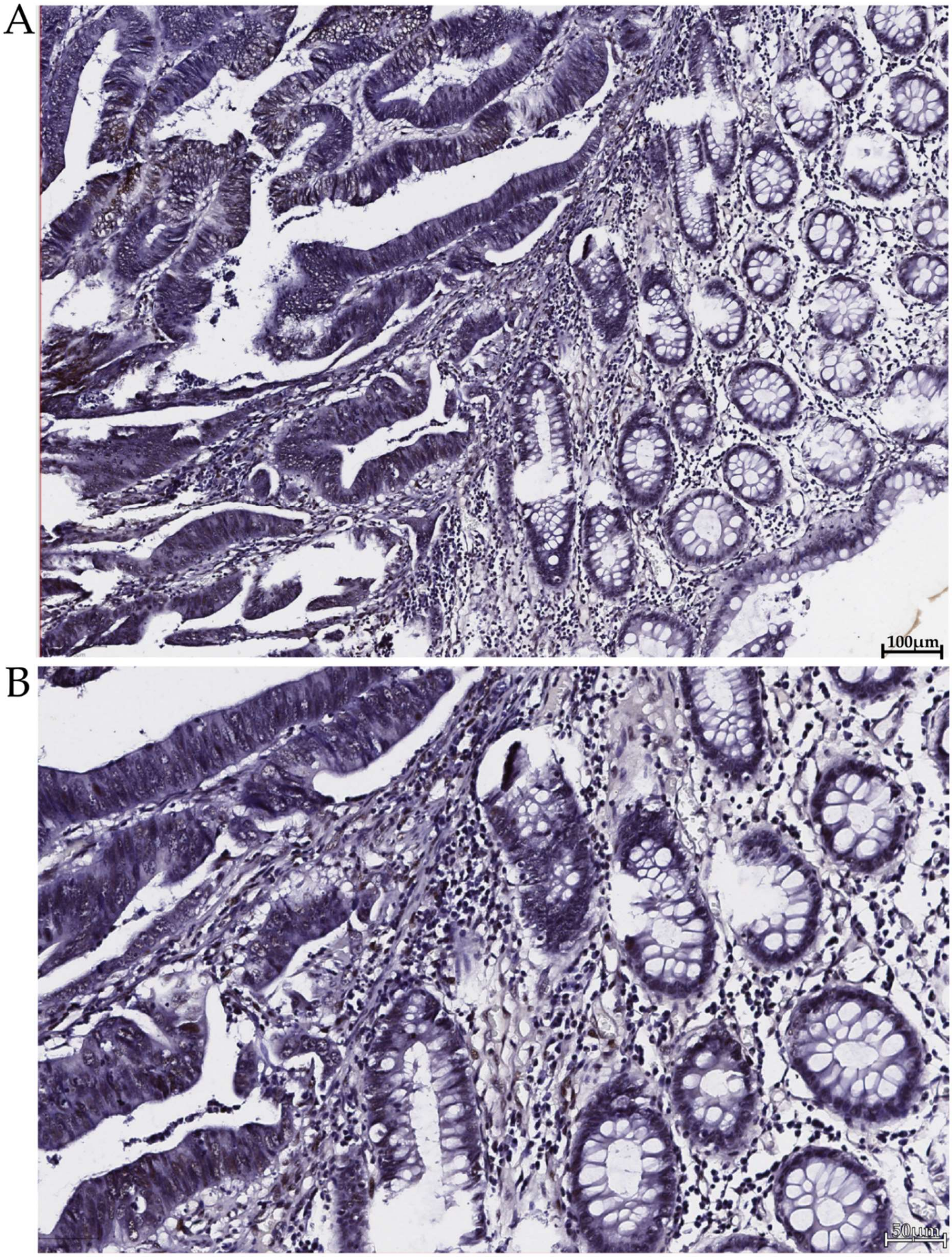

Figure S2. Supplementary Figure S2. PFKFB3 expression is minimal in adjacent normal colonic mucosa. Representative immunohistochemical images demonstrating the specificity of PFKFB3 upregulation in the tumor stroma. **(A)** Tumor area from a colorectal cancer specimen showing stromal PFKFB3^{+} cells (brown DAB). Scale bar = 100 μm **(B)** An area of adjacent, histologically normal intestinal mucosa from the same patient resection specimen, showing minimal to absent PFKFB3 staining. This internal negative control highlights the tumor-associated nature of the PFKFB3 expression observed in the study. Nuclei are counterstained with hematoxylin (blue). Scale bar = 50 μm.
